# An Automated Microfluidic Platform for In Vitro Raman Analysis of Living Cells

**DOI:** 10.3390/bios15070459

**Published:** 2025-07-16

**Authors:** Illya Klyusko, Stefania Scalise, Francesco Guzzi, Luigi Randazzini, Simona Zaccone, Elvira Immacolata Parrotta, Valeria Lucchino, Alessio Merola, Carlo Cosentino, Ulrich Krühne, Isabella Aquila, Giovanni Cuda, Enzo Di Fabrizio, Patrizio Candeloro, Gerardo Perozziello

**Affiliations:** 1 Department of Experimental and Clinical Medicine, University of Catanzaro, Germaneto, 88100 Catanzaro, Italy; stefania.scalise@unicz.it (S.S.); francescoguzzi@aol.it (F.G.); simo.zaccone@gmail.com (S.Z.); parrotta@unicz.it (E.I.P.); valeria.lucchino@unicz.it (V.L.); merola@unicz.it (A.M.); carlo.cosentino@unicz.it (C.C.); isabella.aquila@unicz.it (I.A.); cuda@unicz.it (G.C.); enzo.difabrizio@polito.it (E.D.F.); patrizio.candeloro@unicz.it (P.C.); 2 Department of Chemistry and Biochemical Engineering, Technical University of Denmark, DK-2800 Kgs. Lyhgby, Denmark; ulkr@kt.dtu.dk; 3 Department of Applied Science and Technologies (DISAT), Politecnico di Torino, Corso Duca degli Abruzzi 24, 10129 Torino, Italy

**Keywords:** microfluidics, microfluidic screening devices, in vitro culturing, cell culturing, mini-incubator, cancer cells, Raman spectroscopy, optical imaging

## Abstract

We present a miniaturized, inexpensive, and user-friendly microfluidic platform to support biological applications. The system integrates a mini-incubator providing controlled environmental conditions and housing a microfluidic device for long-term cell culture experiments. The incubator is designed to be compatible with standard inverted optical microscopes and Raman spectrometers, allowing for the non-invasive imaging and spectroscopic analysis of cell cultures in vitro. The microfluidic device, which reproduces a dynamic environment, was optimized to sustain a passive, gravity-driven flow of medium, eliminating the need for an external pumping system and reducing mechanical stress on the cells. The platform was tested using Raman analysis and adherent tumoral cells to assess proliferation prior and subsequent to hydrogen peroxide treatment for oxidative stress induction. The results demonstrated a successful adhesion of cells onto the substrate and their proliferation. Furthermore, the platform is suitable for carrying out optical monitoring of cultures and Raman analysis. In fact, it was possible to discriminate spectra deriving from control and hydrogen peroxide-treated cells in terms of DNA backbone and cellular membrane modification effects provoked by reactive oxygen species (ROS) activity. The 800–1100 cm^−1^ band highlights the destructive effects of ROS on the DNA backbone’s structure, as its rupture modifies its vibration; moreover, unpaired nucleotides are increased in treated sample, as shown in the 1154–1185 cm^−1^ band. Protein synthesis deterioration, led by DNA structure damage, is highlighted in the 1257–1341 cm^−1^, 1440–1450 cm^−1^, and 1640–1670 cm^−1^ bands. Furthermore, membrane damage is emphasized in changes in the 1270, 1301, and 1738 cm^−1^ frequencies, as phospholipid synthesis is accelerated in an attempt to compensate for the membrane damage brought about by the ROS attack. This study highlights the potential use of this platform as an alternative to conventional culturing and analysis procedures, considering that cell culturing, optical imaging, and Raman spectroscopy can be performed simultaneously on living cells with minimal cellular stress and without the need for labeling or fixation.

## 1. Introduction

In vitro cell cultures represent one of the most used methods for scientific research and biological studies. They are used for investigating various aspects of cellular biology, including drug screening [1,2,3], tissue engineering [4,5,6], carcinogenesis [7], cellular aging [8], etc.

Biochemical screening has various aims. It is useful, for example, for investigating the phenotypic, biophysical, and biochemical characteristics of a tumor cell and identifying differences compared to a healthy cell. Hashemzadeh et al. [9] proposed a deep learning algorithm to analyze and classify images of lung cells using algorithms able to automatically recognize malignant cells; other examples can also be found in the literature [10,11,12]. Cell screening can also be used to investigate the presence of macromolecules of interest, like lipids, nucleic acids, proteins, carbohydrates, etc. [13,14,15,16]. Significant advances have been made in high-throughput screening of antibodies to overcome the tedious screening processes of hybridoma technologies and antibody engineering technologies. Lai et al. [17] reported a compact disk (CD) microfluidic device that controls the rotation speed to automatically perform ELISAs for rat immunoglobulin G (IgG) identification. The flow sequence of different solutions in the process was adjusted using centrifugal and capillary forces. Adler et al. [18] used an emulsion droplet microfluidic system combined with an antibody engineering method to screen the antibody repertoire of millions of individual B cells.

One of the key applications of cell screening involves studying the different interactions that can occur between samples and various drugs. Such an approach can assist in pinpointing the most effective drug for treating a specific condition and in understanding the varying responses of patients to different drugs. It is widely acknowledged that not all patients with the same condition react similarly to the same treatment. Thus, drug screening within microfluidic devices is valuable in advancing the personalized medicine approach.

In vitro disease models using human or animal cells can be utilized to study and test treatments when human experimentation is not possible or unethical. The use of in vitro models reduces time, costs, and the need for laboratory animals, aligning with the ethical guideline principles for animal tests known as the 3Rs (replacement, reduction, refinement) [19]. Unfortunately, an evident gap exists between in vitro and in vivo analysis in terms of the effective and realistic information obtained; therefore, new methods which reduce this gap are needed. One critical aspect is related to the effects ON the cell phenotype. Many times, biological samples, and cells in particular, are pretreated before being analyzed. For instance, in fluorescence microscopy, cells are labeled with fluorescent markers or require fixation techniques [20,21]. Although one of the main methods of diagnostic investigation in the clinical evaluation of various pathologies is based on microscopic techniques [22,23], efforts have been made to improve drug screening using spectroscopic techniques [24,25]. In the literature, it is possible to find some examples based on the application of FT-IR [26] or IR-spectroscopy [27] coupled with microfluidic devices and used to screen molecular kinetics. However, these methods are not very adaptable to the aqueous media in which the cells are usually placed, as it is required that cells are positioned as a thin layer immerged in a thin film of medium to partially compensate for the strong IR signal of water for its baseline subtraction [28]. Moreover, more advanced IR spectroscopy techniques exist, such as attenuated total reflection (ATR) FT-IR, which allows us to directly interrogate samples laying on an internal reflection element (IRE)/ATR crystal [29]. Nonetheless, such systems require a complex integration of microfluidic devices built upon an IRE, making the miniaturization of the devices more challenging. Raman spectroscopy is a suitable method for improving such investigations due to its speed, non-invasiveness, and label-free analysis [30,31,32,33]. Nevertheless, Raman spectroscopy usually requires cells to be fixed through specific biological procedures [34,35,36]. In fact, these procedures affect the cell phenotype and kill cells; therefore, it becomes impossible to dynamically follow the changes in a cell during a specific screening analysis.

Another crucial aspect is related to the analysis of cells in a dynamic physiological environment in which they can be analyzed in a time lapse. In fact, many conventional procedures often require taking samples from their static environment to take photographs of a particular state; hence, there is not any possibility to study the kinetics of a biological process. In this regard, microfluidics can help. Unlike traditional methods, microfluidics enables precise control of the cellular microenvironment. Miniaturized systems offer many advantages, like portability, optimal flow control, reduced analysis time, use of small quantities of samples and reagents, reduced production of chemical wastes, and a simple implementation of parallelized processes [37,38]. Bearing these considerations in mind, it follows that cellular screening using microfluidics offers numerous benefits, including higher throughput, precise control of cell manipulation, and the ability to create physiologically relevant microenvironments.

The advantages of a microfluidic culture system with respect to traditional culture methods have been widely discussed in the literature [39,40], allowing for analysis down to single-cell resolution [41,42]. For this purpose, a variety of choices are available to accomplish precise investigation at the single-cell scale, depending on the research needs. These strategies include (i) microwell and microchamber arrays for a precise, random inoculation of cells inside these structures with high throughput; (ii) droplet-based systems with strategically designed microchannels to take advantage of the shearing force of a fluid to dispel and mix a suspension containing cells; (iii) physical trapping structures inside microchannels to capture cells thanks to hydrodynamic or dielectrophoretic forces with precise matching; (iv) non-contact force devices harnessing optical, acoustic, and magnetic phenomena to manipulate and pair cells individually within microfluidic devices, starting from large sample volumes, while minimizing cell damage [43]. However, a flawless microfluidic device for managing the cellular microenvironment has not yet been developed, since these devices are often complex to use, requiring specialized personnel, while screening procedures are commonly handled by biologists and biotechnologists.

In this paper, we describe an automated, portable, miniaturized passive microfluidic device (PMD) and an on-stage mini-incubator (MI) that can be coupled to optical instruments, such as microscopes and spectrometers, through customized adapters. This setup facilitates the long-lasting, real-time analysis of living cells in a controlled dynamic environment, allowing researchers to examine their morphology and spectra.

Different miniaturized incubator prototypes have been researched and developed over the decades. Vukasinovic et al. [44] developed a compact-sized incubator which contains a microfluidic perfusion chamber. The system is equipped with temperature sensors, with the possibility of integrating, according to needs, pH, glucose, and oxygen sensors. However, this system cannot be coupled with microscopy/spectroscopy instruments, as it lacks optical windows, and it is not equipped with a humidification mechanism nor UV LEDs for sterilization. Yamashiro et al. [45] and Resch et al. [46] used commercially available incubators developed by Tokai Hit and coupled them with inverted optical microscopes; other examples can also be found in the literature [47,48,49,50,51]. Although microfluidic platforms capable of controlling the cellular microenvironment are available on the market, they do not always contain all the necessary tools, such as sterilization systems or specific sensors.

We developed a microfluidic platform specifically designed to reproduce a dynamic microenvironment which passively drives liquids and in which samples and reagents are handled by procedures conventionally used by biologists. The platform is composed of a disposable PMD integrated into an MI, which can be mounted and coupled to optical microscopes and Raman spectroscopy instruments. The platform was used to grow cultures of cells, induce oxidative stress, and, after 24 h, perform Raman measurements. Through the investigations, it was possible to achieve biochemical discriminations of control cells and oxygen peroxide-treated cells in vitro, proving the ability of our microfluidic platform to perform real-time biological investigations exceeding the limit of conventional analysis techniques, such as cell fixation or invasive cell labeling with dyes, which cause cell disruption, preventing further investigation.

## 2. Methods

### 2.1. Materials

#### 2.1.1. PMD

Polymethyl methacrylate (PMMA) was purchased from Röhm Italia SRL, Settimo Milanese, Italy. DESKAM 2000 version 5.1.5.11 was provided by the Minitech Machinery Corp, Norcross, CA, USA. End-mills were purchased from Performance Micro Tool, Performance Micro Tool, USA. Absolute anhydrous ethanol was purchased from Sigma-Aldrich, St. Louis, MI, USA. Raman-grade CaF_2_ slides were purchased from Crystran, Poole, UK. A white polyester biocompatible double-sided tape (100 μm thick) was purchased from Adhesive Research US, Glen Rock, PA, USA.

#### 2.1.2. MI

Model SLA Form 3+ was purchased from Formlabs, Somerville, MA, USA. PLA was purchased from FILOALFA-ALFAPRO, Turin, Italy. Grey Pro resin was obtained from Formlabs. Water-soluble PVA was obtained from Raise3D Premium. Cura software version 5.4 and ideaMaker software version 4.2.3 were purchased from their respective sellers.

Heating glass, featuring built-in Indium tin oxide, was purchased from Yalos–Glass Technology. Hot-end heating blocks for 3D printers were obtained from MakerHawk. The DS18B20 temperature sensor was obtained from Dallas Semiconductor. K-type thermocouples were purchased from RS Pro. A sliding sterilization lid was designed and mounted with 6 UV-LEDs, model LEUVA66B00HF00, produced by LG Innotek, at 278 nm. Ni60/Cr16 conductive wire with a declared resistance of 6.9 Ω/ft was obtained from Omega, Stamford, Connecticut. A West 2300 PIC external controller was purchased from RS Pro. A cylinder with a gas mixture of 20% O_2_, 5% CO_2_, and 75% N_2_ was obtained from I.C.O.A. srl. A capacitive touch sensor, MPR121, produced by Adafruit (Brooklyn, NY, USA), was used. A temperature controller model TC-324B was obtained from Warner Instruments (Holliston, MA, USA). IPS-2303 laboratory DC power supply was obtained from ISOTECH (Colchester, VT, USA). An Ansys Workbench 2021 R1 simulation license was obtained, comprising CFX and Fluent solvers.

An overview of other fundamental components can be found in Supplementary Table S1.

#### 2.1.3. Biological Samples and Reagents

HeLa and HEK293T cell lines were obtained from the American Type Culture Collection (ATCC, Manassas, VA, USA). HeLa cells were cultured in Roswell Park Memorial Institute (RPMI) 1640 medium (Corning, Corning, NY, USA), while HEK293T cells were maintained in HyClone™ DMEM/High Glucose Modified medium (General Electric, Boston, MA, USA). All culture media were supplemented with 10% fetal bovine serum (FBS) and 1% penicillin–streptomycin. Cells were incubated at 37 °C in a humidified atmosphere containing 5% CO_2_.

#### 2.1.4. Raman Data Analysis

RamanToolSet analysis software version 2.3.2 was developed by our research group.

### 2.2. Fabrication of the PMD

Micro-milling was chosen for the fabrication of the PMD. Although a variety of techniques are available [52,53], micro-milling meets most of the specific requirements of our study. This technique is low-cost and allows for high precision, reproducibility, and fast prototyping, with a high degree of design freedom—e.g., complex channel structures can be easily achieved using commercially available end-mills with different geometries (square, rectangular, circular, semicircular, etc.). Compared to other techniques, such as laser engraving, photolithography, or replica molding, micro-milling offers several practical advantages. Laser engraving is faster but limited to shallow 2D profiles and may cause thermal damage on thermoplastics. Photolithography ensures high resolution and smoothness but requires cleanroom facilities and costly photomasks. Replica molding using PDMS provides excellent fidelity but depends on a master mold and may involve issues like hydrophobicity, gas permeability, and molecule absorption, which can limit material compatibility. In contrast, micro-milling enables direct structuring of thermoplastics (e.g., PMMA, COC) with acceptable surface roughness (typically in the 0.1–2 µm range [54,55,56] and good chemical stability, making it suitable both for prototyping and for small-scale production.

**Figure 1:**
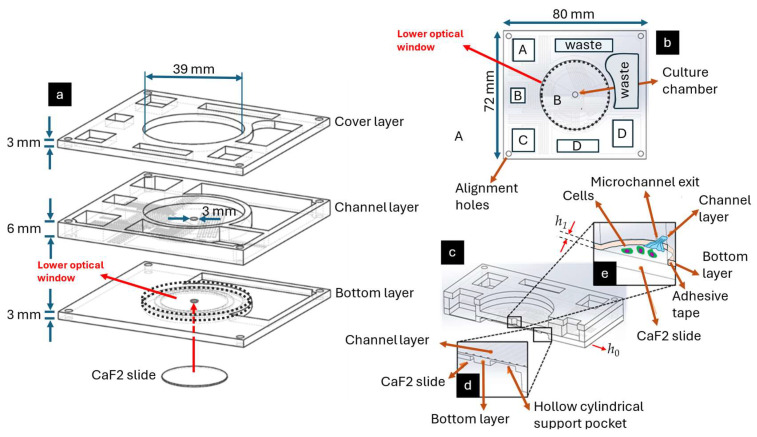
Three-dimensional scheme of the PMD. (**a**) Representation of the three layers of the PMD: (i) a cover layer was machined on both faces; the internal face has openings that let the liquids pass through, and the external face has a large central optical window manufactured after the bonding process; (ii) a channel layer was machined on both faces; the bottom face has 5 microchannels, while the top face has a large optical central window manufactured after the bonding process; (iii) a bottom layer was machined on both faces; the upper face has a milled 2.7 mm deep tank which will create the collection tank (waste); at the center, there is an opening for cell culturing, which is closed on the bottom by attaching a CaF_2_ slide; the slide is attached inside a milled pocket after the bonding process. It is worth highlighting that the bottom and the upper circular optical openings allow the microscope objective to approach the cell culture in both direct and inverted configurations. (**b**) Upper view of the device with its media input reservoirs A, B, C, D, waste reservoir, and the upper optical window. (**c**) Cross-sectional view of the device, namely the cells in the central reservoir, which have to be fed by the media carried by the microchannels; below the cell culture’s reservoir, there is a CaF_2_ slide attached. (**d**) Cross-sectional detail of the CaF_2_ slide’s positioning with respect to the microchannel plane. The adhesive tape is positioned between the bottom layer and the CaF_2_ slide. (**e**) Schematic view of the cells lying on a plane 100 μm below the microchannel plane because of the adhesive tape’s thickness, denoted with h_1_.

The Mini-Mill/GX micro-milling machine model was used to manufacture the microfluidic device in the present study. Plastic layers were made of 3 mm and 6 mm thick PMMA. Raw PMMA sheets were securely fixed onto the micro-mill’s working plane, and they were micro-milled. The spindle was controlled by a Nakanishi E300C controller. FreeCAD 3D was used to create 3D models of the layers (Figure 1). DESKAM 2000 software (V2.0) was used to generate the G-codes for the milling process. End-mills 0.25 mm, 0.5 mm, 1, mm, 2 mm, and 3 mm in diameter were used. All channels had a rectangular shape with dimensions of 0.25 mm × 0.10 mm × 300 mm (width × height × length). Post-fabrication cleaning was performed via washing with deionized water and soap and then isopropanol immersion for 2 min; finally, the layers were dried with nitrogen. PMMA layers were subsequently assembled with the solvent-assisted bonding technique by using absolute anhydrous ethanol and a P/O/Weber Laborpresstechnik column lab manual press.

After the assembly of the device, two optical windows with a diameter of 39 mm were milled on the top and bottom layers (Figure 1a,b) to allow the microscope’s objectives to approach the biological samples inside the central culture chamber in both direct and inverted optical configurations. PMMA is not suitable for cellular analysis with Raman techniques due to autofluorescence phenomena that would interfere with signals coming from biological components. Therefore, a Raman-grade CaF_2_ slide with a diameter of 25 mm was secured onto a specialized pocket created beneath the culture chamber (Figure 1d) using a 100 μm thick biocompatible double-sided adhesive tape, serving as the substrate for cell growth and proliferation (Figure 1e).

### 2.3. Fabrication of the MI

FreeCAD version 0.21 3D was used for the CAD design of the MI (Supplementary Figure S1). Micro-milling and 3D printing were used to manufacture the MI. Transparent surfaces such as lateral walls and holders for the heating glass were made of 6 mm thick PMMA via micro-milling.

Three-dimensional printers (FDM double nozzles) were used to manufacture components with complex shapes and designs, such as the microscope stage adapter, water tank, heating block holders, etc. Cura software and ideaMaker software were used for the slicing process of 3D printing. Screws, glue, double-sided tape, and electrical wires were used during the assembly.

Ansys Workbench 2021 R1 was used to perform finite element simulations (FEM) and investigate the temperature control and distribution in the MI. A detailed overview of the simulations conducted on the MI is described in Supplementary Chapter S1. A heating glass and two heating blocks were used to heat up the water, each one connected to a waterproof K-type thermocouple, except for the heating glass, whose temperature was monitored via a digital temperature sensor (Supplementary Figure S1a). A custom-made cart heater was created using a nichrome conductive wire.

### 2.4. Working Principles

**Figure 2:**
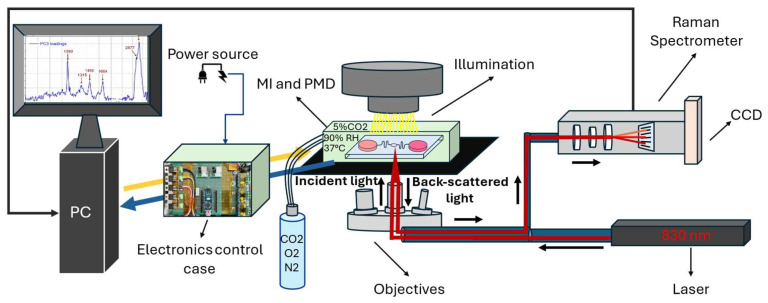
The microfluidic platform, consisting of the MI and PMD, is connected to the external dedicated PID controller, powered by an external generator, which monitors the cart heater temperature thanks to a K-type thermocouple. For CO_2_ supply to the system, a cylinder with a gas mixture of 20% O_2_, 5% CO_2_, and 75% N_2_ is used. The assembled MI was coupled to an inVia Raman spectrometer from Renishaw, Gloucestershire, UK, or to an Eclipse-Ti inverted microscope (Nikon Instruments Inc., Melville, NY, USA), depending on the experiments. An IR laser is utilized in this setup. A graphic user interface developed in Python 3.12 is used to monitor and set the parameters. A water level sensor is utilized to trace the water level in the MI’s internal humidity tank.

The microfluidic platform use case is represented in Figure 2. The PMD is positioned inside of the MI described in this work. The MI is coupled to a case built to house all the electronics for the control and monitoring of the incubation parameters, such as temperature, relative humidity, and % of carbon dioxide (Table 1). The MI is compatible with microscope stages to be suitable for coupling to any type of inverted microscope. The various parameters can be set up and monitored via a touch screen or from a computer. Concerning our passive microfluidic systems, they are open systems which can be accessed from above for the insertion of the various reagents and biological samples or, on the contrary, to take them for subsequent analysis using conventional pipettes, so this alone makes them simpler to use and familiar even to non-technician operators, for instance, biologists. Furthermore, the liquids inside the device move passively, namely, there are no active actuators to create pressure gradients. In fact, liquids move inside the device due to gravity through a system of communicating vessels, a concept explained in Figure S2.

The biological sample is placed in a chamber that integrates an optical window allowing for the analysis of cells through the coupled microscope in real time through various techniques, including, for example, Raman spectroscopy. The sample can be monitored in real time, and this is a peculiarity that is not present in common incubators.

#### 2.4.1. Operating Principles of PMD

The PMD was used for housing tumoral cell cultures. It has four inlet reservoirs and a waste reservoir. The microfluidic network is composed of four inlet reservoirs, namely, A, B, C and D; five channels, each connected to a cell culture reservoir; and one single outlet channel (waste). It is worth specifying that the presence of multiple inlet reservoirs is due to the design robustness of the PMD, in order to allow for more degrees of freedom concerning biological protocols; it means that the PMD can be used with only one inlet reservoir if only one medium is sufficient, and it can also be used with up to four inlet reservoirs along with four different media if the biological protocol requires it.

The system is designed to have the surface on which proliferating cells adhere located 0.200 mm below the microchannel openings to prevent cells from being subjected to high shear stresses that could directly affect their proliferation. Different reservoirs are designed to have varying volume capabilities, based on how many days they should feed the culture chamber. To prevent the return of the waste liquid back to the culture reservoir, the waste reservoir was manufactured on the internal surface of the bottom layer at a lower level compared to the culture chamber and other reservoirs, hence creating a tank.

From a hydrodynamic point of view, the device works with no additional external pressure sources. Namely, with a proper dimensioned hydraulic resistance, a difference in the heights of the liquid levels gives rise to a flow rate in the microchannels, as the Stevino law states.

A crucial part of the design is based on the correct dimensioning of the microchannels in terms of their microfluidic resistance, R; it considers the type of medium, which is characterized by its own viscosity and the geometry of the channel [57].

#### 2.4.2. PMD’s Flow Rate Measurement

Before the actual utilization of the PMD for cell culturing, each channel’s steady-state flow rate was evaluated. Given the four inlet reservoirs of the PMD, namely A, B, C, and D, each of them was assessed in terms of the steady-state flow rate. A protocol allowing for inlet-to-cell-to-waste reservoir connection was developed. This allowed us to create focused temporary hydraulic circuits to allow for the microchannel testing of single pairs. The protocol is illustrated in Supplementary Figure S5.

#### 2.4.3. Operating Principles of MI

The MI integrates three main systems for heating, humidification, and sterilization. Each system is controlled by a PID controller:

1. Heating of the MI allows it to maintain the target temperature of the environment. This was achieved by the transparent conductive glass attached to the sliding lid, two 3D printer hot-end heating blocks placed inside the MI, and a custom-made donut-shaped Joule-effect heater placed onto the sliding drawer on which the PMD lays;
2. A humidification system was used to achieve high relative humidity inside the MI environment in order to mitigate the evaporation rate of the media of the PMD. Two 3D printer hot-end heating blocks were placed inside the water tank inside the MI to make the water evaporate. A water level sensor was used to ensure water was not missing inside the tank, and, as it evaporated, a volumetric pump refilled the water tank to compensate for the evaporation;
3. Sterilization was achieved via 6 UV-LEDs installed onto the sliding lid. The number of LEDs was determined based on the area that needed to be sterilized. The exposure time estimation (circa 30 min) was based on the UV dose needed for the biological inactivation of bacteria (Supplementary Table S4). Prior and subsequent to the biological experiments, sterilization was carried out.

Each system was controlled by a Teensy 3.2 microcontroller. A PID control Arduino code was written to allow the microcontroller to communicate with each component, as well as monitor and control them in order to maintain the desired parameters. A GUI was developed to allow the operator to input values using a touch screen. Additional information can be found in Supplementary Chapter S2.

### 2.5. Experimental Setup

Once assembled, the MI was connected to the Renishaw inverted Raman spectroscope. The water tank was filled with approximately 60 mL of water. The microcontroller was linked to the PC via the USB port, and the user initiated the graphic interface. Afterwards, the system was powered with a benchtop power supply generator in parallel configuration, providing a total output of 12 V and 3 A. The user typed the desired values via the GUI, prompting the MI to begin heating the heating blocks inside the water tank and the heating glass. An additional heater was placed beneath the sliding cart. This was necessary to minimize heat loss from the aperture in the middle of the MI, which served as an optical window for positioning the microscope objectives near the samples. The cart heater glued beneath the sliding cart. A thermocouple was affixed to the heater’s surface to monitor and measure the temperature once it was connected to the dedicated external West 2300 controller.

### 2.6. Biological Experiments

The cell culture procedure within the microfluidic platform was carried out as follows: First, all reservoirs (A, B, C, D, and waste) were filled with the appropriate volumes of culture medium, as specified in Table S2. To ensure complete filling of the microchannels and to eliminate air bubbles, negative pressure was applied through the central culture chamber using a syringe fitted with a vented tip.

Once the channels were properly filled, all inlet reservoirs were sealed with sterilized adhesive tape to eliminate undesired pressure-driven flow into the culture chamber. Residual liquid in the chamber was then removed, and the waste reservoir was emptied.

Cells were subsequently seeded in the central culture chamber by introducing 15 μL of culture medium containing 62,500 cells/mL. The system was left undisturbed for 15 min to allow the cells to settle at the bottom of the chamber. Following this step, the adhesive tape was selectively removed only from the reservoir intended for active flow generation.

To promote wetting of the outlet microchannel and overcome hydrophobic barriers, 200 μL of culture medium was added at the entrance of the waste reservoir. Finally, the complete microfluidic setup was placed inside a large sterile Petri dish and transferred to a conventional (or dedicated) incubator for subsequent culture and analysis.

HeLa cells were cultivated for 72 h with the RPMI culture medium to test the functioning of the PMD. Cell proliferation was evaluated considering images related to three different regions of interest (ROIs) every 24 h.

Once the behavior of the platforms was assessed, the MI was sterilized by placing its lid on for 30 min. Subsequently, the cylinder containing a gas mixture of 20% O_2_, 5% CO_2_, and 75% N_2_ was then connected to the MI through the dedicated air hole. Cells were then seeded onto our dedicated microfluidic devices and cultured for 24 h with the same parameters, as shown in Table 1. Two separate culturing experiments were conducted using the same protocol, with each experiment utilizing a subset of cells derived from the same cellular split. The first experiment served as the control set and was carried out normally, while the second experiment involved a hydrogen peroxide (H_2_O_2_) treatment with a concentration of 5.09 mM.

### 2.7. Experimental Characterization

To simulate the real experimental conditions, a microfluidic device was housed in the MI without any biological samples. The presence of the culture support inside the sliding cart prevented moist air from escaping through the optical window located at the bottom of the MI, thereby increasing the relative humidity inside the culture environment. After determining the ideal values for each heat source (Table 2), a preliminary analysis of temperature and humidity profiles for a period of 2 h was carried out.

### 2.8. Experimental Procedure with Biological Samples

At the conclusion of the biological experiment, the Raman spectra of the cells under investigation were acquired as maps by means of Renishaw’s inVia spectroscope. An IR laser at 830 nm wavelength was exploited for the collection of the Raman back-scattered signal, which was detected by the inVia spectroscope. Two maps were acquired, one for the HeLa control sample and the other for the H_2_O_2_-treated HeLa sample, aimed at inducing oxidative stress and investigating any resulting differences in the Raman spectra. Moreover, the phenol red-free medium was used for cell culturing so as not to interfere with the Raman signal due to autofluorescence phenomena. The specific details of the two maps are provided below:

- The first map was collected from the control sample using an objective lens with a magnification of 50× and N.A. 0.50. The acquired map had dimensions of 32 μm × 36 μm. The pixel matrix used was 16 × 18, resulting in a total of 288 pixels. Each pixel had a square shape with a side length of 2 μm.
- The second map was acquired from the H_2_O_2_-treated sample using the same 50×/0.50 objective lens. The dimensions of this map were 22 μm × 22 μm. The pixel matrix used for this map was 11 × 11, resulting in a total of 121 pixels. Each pixel had a square shape with a side length of 2 μm.

Each cell map was preprocessed by subtracting its own average spectrum of the medium. Hence, each cell map was considered without its surrounding medium; otherwise, it would interfere with the subsequent analysis. The maps were subjected to topological and differential Raman analysis using RamanToolSet via the following protocol: (i) the two maps were spatially combined and treated as a single map representing two different regions. To achieve this, the coordinates of the treated sample were shifted by a certain amount along the X and Y axes; (ii) removal of cosmic rays; (iii) baseline subtraction using a fourth-order polynomial; (iv) area normalization based on the maximum peak area to allow for comparison between measurements associated with the two samples; (v) performing PCA analysis; (vi) applying K-mean clustering with four classes corresponding to the membrane, cytoplasm, nucleus, and background.

## 3. Results

**Figure 3:**
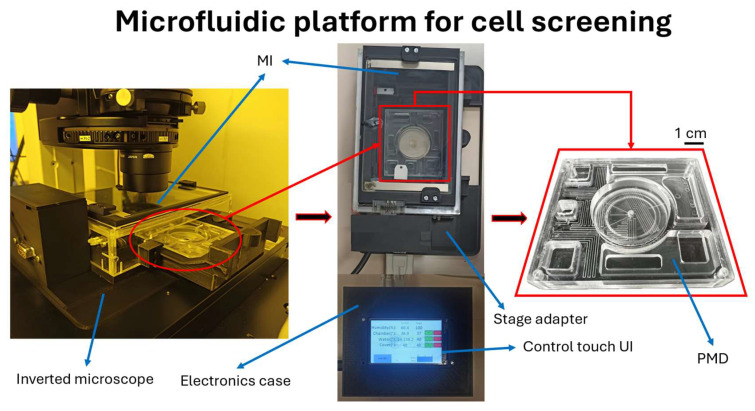
(**left**) The resulting microfluidic platform composed of the MI and PMD prior to its working conditions; (**center**) top view of the platform inside the MI along with the touch screen with dedicated electronics and GUI allowing for the setting and monitoring of incubation parameters; (**right**) close-up view of the PMD used in this work.

A photograph of the microfluidic platform, positioned on the Nikon Eclipse-Ti inverted microscope’s stage, is shown in Figure 3. After assessing the functioning of both the PMD and MI, the PMD was charged with the culture media and inserted into the already operating MI along with its dedicated electronics. The platform was utilized with the Renishaw inVia spectroscope and Eclipse-Ti Nikon inverted microscope by positioning it upon the stages for cell culturing and its assessment. The obtained data was then processed, analyzed, and interpreted.

### 3.1. Experimental Characterization of the PMD

The flow rate of the PMD was determined and compared to the theoretical values. By referring to Supplementary Table S2 and replacing the value of reservoir D, the theoretical hydraulic resistance was determined to be equal to $R_{H}=1.92\cdot 10^{13}\;Pa\;s\;m^{-3}$ (Supplementary Chapter S3). By applying the Huygens–Poiseuille law, the theoretical flow rate, Q, was determined to be circa equal to $11.2\;µL\;h^{-1}$.

To determine the effective flow rate, 200 μL of distilled water was placed in the exit of the waste reservoir’s channel in order to overcome the surface tension between the medium and PMMA and let the medium flow out of the channel. After 4 h, the total volume collected was 227 μL/h; hence, the flow rate was 6.7 μL/h, while, theoretically, it should have been equal to 11.2 μL/h. This difference is to be regarded as the result of the manufacturing process in terms of channel geometry, wherein the cross-sectional height fabrication tolerance of ±30 μm, which directly affects the final flow rate, should be considered; indeed, this tolerance depends on the cube of the smallest side of the cross-section of the microchannel. Moreover, the flow rate difference is to be found in the evaporation rate of the volume laying in the waste reservoir before being collected.

The layout configuration of the PMD allowed us to create a semi-static environment for the cells. In fact, cell culture was placed in the 100 μm high pocket of the culture chamber. The height of this pocket is given by the thickness of the adhesive (see Figure 1e). This reproduced a dynamic, blood microcirculation-like environment, where the exchange of substances between blood and cells occurs as a consequence of the small volumetric flow of the media above the cells. In addition, this configuration prevents the flows from flushing the cells out of the culture chamber.

**Figure 4:**
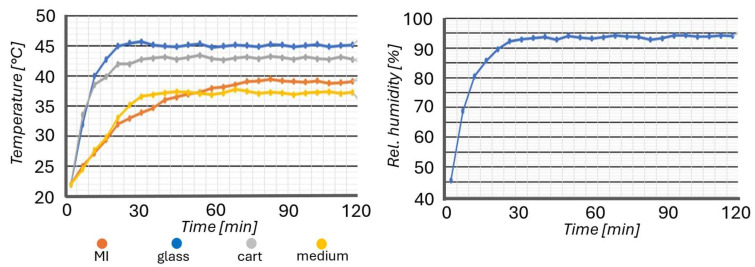
Experimental temperature (**left**) and humidity (**right**) profiles recorded for 2 h.

The temperature and humidity profiles recorded inside the miniaturized incubator (MI) over a 2 h period prior to the biological experiments, in order to determine the optimal parameters of the heating elements, are shown in Figure 4. A thermocouple was immersed inside the cell culture-dedicated reservoir with no culture inside of it. The functioning of the different heating components was set in a manner that resulted in an optimal heating of the culture medium of circa 37 °C and optimal relative humidity, with no dangerous heating ramps.

### 3.2. Experimental Validation with Biological Samples

**Figure 5:**
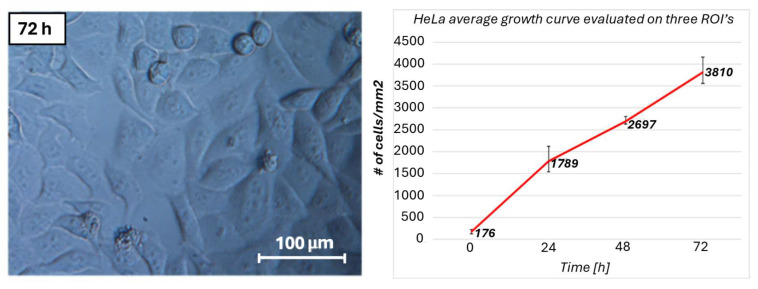
Microscope photograph of the resulting culture after 72 h (**left**); growth curve of HeLa cells during the PMD functioning assessment at different time periods(**right**).

HeLa cells exhibited a good adhesion over the CaF_2_ slide. Furthermore, cell proliferation was not affected by the combination of the hydrodynamic parameters used. Cells notably increased in number and reached confluence in 72 h, as shown on the right of the chart in Figure 5. The trends are comparable with the results presented in the literature [58]. An optical image of the cells after 72 h is shown on the left of Figure 5.

**Figure 6:**
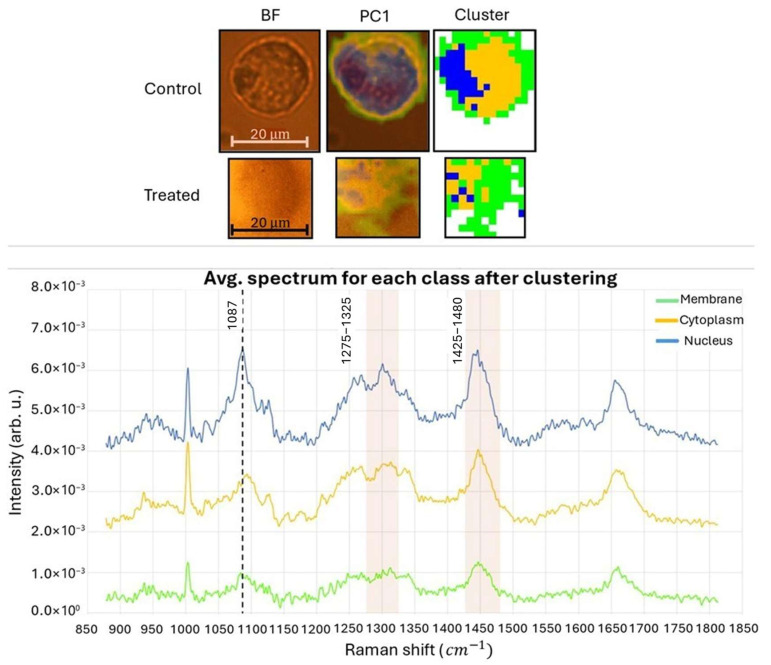
The BF, PC1, and clustering images are shown for each sample. It can be seen how cellular compartments are well-clustered in the control sample, unlike in the treated cell. In the cluster maps, the white color is related to the background, while the green, yellow, and blue colors refer, respectively, to the external membrane, cytoplasm, and nuclear region. The latter seems to be very fragmented in the treated sample (**top**). Average spectrum calculated for each cluster, considering the signal coming from both the samples. Note how the signals associated with the nuclear and cytoplasm cluster are the most significant (**bottom**).

The results obtained from the topological Raman analysis of cells can be seen in Figure 6. For each sample, from left to right, the optical images acquired in bright field and the corresponding PC1 images are shown, along with the clustering images. It is evident that the cellular compartments have been effectively clustered in the control sample, whereas this is not the case in the treated cell. In the cluster maps, the background is represented by the white color, while the external membrane, cytoplasm, and nuclear region are represented by green, yellow, and blue colors, respectively. It is apparent that the nuclear region in the treated sample appears to be highly fragmented. The nuclear signal associated with the blue class exhibits peaks that are typically related to the DNA backbone (1087 cm^−1^) and its components (bands at 1275–1325 cm^−1^ and 1425–1480 cm^−1^), and these peaks are higher compared to those observed in the other spectra [59,60].

**Figure 7:**
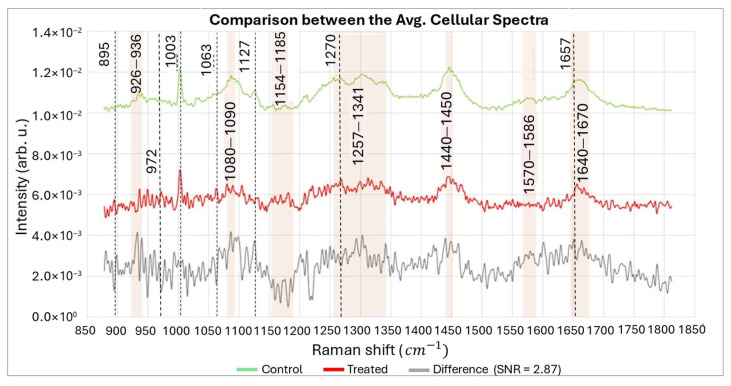
Average cellular spectra of the nucleus and cytoplasm (respectively, the yellow and blue classes in Figure 6) for the control and treated samples. The resulting spectra are smoothed and plotted together to perform a differential analysis. The difference spectrum is calculated by subtracting the treated sample from the control. The signal-to-noise ratio (SNR) is 2.87. Noise was evaluated on the difference spectrum, and it was compared with the most significant Raman bands. The resulting difference spectrum is amplified by a 2.5 factor.

As previously mentioned, the spectra of each class related to the membrane (green class), cytoplasm (yellow class) and nucleus (blue class) were joined and averaged (Figure 6, bottom). Figure 7 displays the smoothed and plotted spectra obtained to conduct a differential analysis. Table 3 presents the potential assignments for the Raman peaks. The Raman bands within the 800−1100 cm^−1^ range are commonly affected by DNA backbone geometry and secondary structure [61]. By referring to Figure 7, it can be observed that the bands associated with DNA backbone vibrations (926−940 cm^−1^ and 1080−1090 cm^−1^) are modified in the treated sample. The removal of hydrogen atoms from the sugar phosphate backbone by ROS leads to DNA damage, causing the destabilization of the DNA duplex and resulting in the breakage of hydrogen bonds between base pairs. This process generates multiple unpaired base residues [62].

These findings may account for the enhanced peaks observed in the 1154–1185 cm^−1^ region of the treated sample, potentially arising from unpaired nucleotide bases [59,61,63]. Furthermore, damage to the DNA structure disrupts protein synthesis, leading to a decrease in protein content. This decrease is reflected in the intensities observed in the corresponding bands at 1257–1341 cm^−1^, 1440–1450 cm^−1^, and 1640–1670 cm^−1^ [59,61,64,65]. Oxidative stress induces membrane damage, leading to the degradation of the damaged phosphatidylcholine and to the activation of the Kennedy pathway responsible for phospholipid synthesis, including phosphatidylcholine, in an attempt of the cell to repair the damaged membrane [66]. This phenomenon leads to a temporary increase in phosphatidylcholine concentration and, consequently, its Raman signal at 1270, 1301, and 1738 cm^−1^ [67,68].

**Table 3:** Possible assignments of peaks of Raman spectra of cells prior and subsequently to oxidative treatment with 5.09 mM of H_2_O_2_. The increase (↑), decrease (↓), or conservation (↔) of Raman peaks after the treatment are reported at a specific Raman shift in the band of interest.

Before ROS Attack	After ROS Attack	Possible Assignments of Raman Peaks	References
895	↑	Phosphodiester, Deoxyribose	[69]
926–940	↓↑	DNA backbone vibration	[59,70]
972	↑	Phosphatidylcholine	[67,68]
1003	↔	Phenylalanine	[59]
1063	↑	C-C skeletal stretch random conformation	[71]
1080–1090	↓	DNA backbone vibration	[59,61,63]
1127	↓	C-N stretching (proteins) C-O stretching (carbohydrates)	[72]
1154–1185	↑	Unpaired nucleotides	[60,61,63]
1270	↑	Phosphatidylcholine	[67,68]
1257–1341	↓	dA, dT (ring breathing modes of the DNA/RNA bases), Amide III (protein), CH_3_, CH_2_ (twisting), CH_2_ deformation (lipid)	[59,73,74]
1440–1450	↓	CH_2_ bending modes of proteins and lipids	[59,61]
1570–1586	↓	dG, dA, ring breathing modes in the DNA bases, C=C Phenylalanine, Pyrimidine ring (nucleic acids)	[59]
1657	↑	Phosphatidylcholine	[67,68]
1640–1670	↓	Amide I, Nucleic acids, C=O and C=C stretches	[59,64]

## 4. Conclusions

In this work, an automatized, inexpensive, portable, and miniaturized microfluidic platform was developed. It consists of a mini-incubator reproducing a physiological environment through the control of parameters such as CO_2_, humidity, and temperature and a passive microfluidic device for cell culturing. The platform was employed for biological and biochemical assays via Raman spectroscopy and microscopy. Moreover, the platform was controlled by the user via a GUI programmed in Python and a touch screen for the rapid setup of control parameters.

In contrast to conventional incubators, the system is designed as a portable instrument, enabling the real-time monitoring of microfluidic samples without the need for extraction and without exposing the culture to temperature or chemical perturbations. This architecture also facilitates in situ Raman spectroscopy on live samples. Furthermore, the mini-incubator’s portability allows it to be easily adapted to multiple inverted microscopy platforms.

The resulting microfluidic platform was successfully employed for biological experiments. Cell cultures exhibit no sign of stress: the cells adhered to the substrate well and proliferated without evidence of apoptosis. Furthermore, since different reagents can interact with biological samples at specific time points through appropriate microfluidic channel network design, different, more complex and long-term biological experiments may be carried out on the microfluidic platform. This is enabled by the combination of the microfluidic device’s versatility and the autonomous functioning of the integrated mini-incubator.

Raman spectroscopy was carried out on in vitro control and H_2_O_2_-treated samples in order to non-invasively investigate the biochemical changes occurring in living cells. The results suggested that oxidative stress may be recognized in the Raman band of the biochemical fingerprint. In fact, DNA damage occurred as a consequence of backbone rupture, leading to unpaired base dispersion and protein synthesis interruption. In addition, evidence of oxidative stress can be found in membrane damage resulting from phospholipid degradation. This degradation activates cellular pathways aimed at repairing the injury, which leads to the synthesis of new phospholipids and a transient increase in their concentration.

The measures performed are preliminary and will be refined in future work. In fact, the size of the mapped areas limits pixel resolution. Additionally, cells are imaged in living conditions and are not fixed; hence, it is crucial to develop fast analysis techniques. This will help reduce the duration of scanning, as well as minimize stress on the sample. Furthermore, due to cellular movements and micro-vibrations occurring on the motorized stage, a prolonged scanning process is currently impractical without the implementation of high-speed scanning procedures.

Moreover, the use of rapid prototyping components such as a CNC milling machine, milled PMMA microfluidic devices, and an Arduino-based microcontroller offers advantages in terms of development speed, modularity, and flexibility. However, transitioning this system into a scalable, clinically applicable product will require addressing several determinant challenges. In fact, the Arduino controller must be replaced with a custom-designed embedded system based on medical-grade components with documented electromagnetic compatibility (EMC), electrical safety (e.g., IEC 60601-1 compliance [75]), and a robust firmware architecture, in order to guarantee device stability and safety; the manufacturing process must be designed for larger-scale production with higher reproducibility while maintaining biocompatibility, as samples come in direct contact with the PMMA, as required from ISO 10993 [76] standards. Regulatory compliance will also play an important role in future developments. To be marketed as a medical device, the platform will require certification according to ISO 13482 (quality management for medical devices) [77], IEC 62304 (software lifecycle) [78], and CE marking or FDA clearance, depending on the target market. This includes comprehensive risk management, system validation, documentation, and usability engineering in line with IEC 62366 [79].

This platform demonstrates how miniaturized and autonomous microfluidic systems can support advanced cell-based assays and real-time biosensing, setting the stage for future integration in laboratory automation and biomedical research tools.

## Figures and Tables

**Table 1:** Best physical and chemical parameters for culturing human cells.

Optimal Parameters for the Reproduction of the Cellular Microenvironment	
Temperature	≅37 °C
Relative humidity	90%÷95%
pH	7.2÷7.4
$CO_{2}$	3%÷7%
Sterility	Sterile environment

**Table 2:** Temperature parameters used for the experimental characterization of MI.

Parameter	Value
Heating glass temperature	45 °C
Heating blocks temperature	50 °C
Cart heater temperature	43 °C

## Data Availability

Data are available upon request from the corresponding authors.

## Supplementary Materials

The following supporting information can be downloaded at https://www.mdpi.com/article/10.3390/bios15070459/s1, Table S1: Overview of components used for the MI; Table S2: Detailed design dimensions of the PMD; Table S3: Parameters and values used in the simulation; Table S4: Maximum UV-C dose to inhibit different microorganisms; Table S5: Approximative cost of the microfluidic platform; Figure S1: 3D CAD model of the MI; Figure S2: The concept of communicating vessels; Figure S3: Detailed view of the channel layer; Figure S4: Detailed view of the boundary conditions applied to water and air domains; Figure S5: Flow rate measurement protocol; Figure S6: Top closures of the MI; Figure S7: Side views of the MI; Figure S8: Touch screen display fixed onto the top face of the microcontroller case; Figure S9: The assembled case of the control system. References [80,81,82,83].
