# Supplementary material for: An Automated Microfluidic Platform for In Vitro Raman Analysis of Living Cells
Source: Biosensors (Basel). 2025 Jul 16;15(7):459. doi: 10.3390/bios15070459 (PMC12293637; doi:10.3390/bios15070459)
Supplement: Supplementary file 1 [file biosensors-15-00459-s001.zip › biosensors-3704532-supplementary/supplementary_v3.pdf]

Supplementary Materials

Table S1.Overview of other fundamental components used for the MI development.

Description	Component/model	Producer
Microcontroller	Teensy 3.2	Teensy
Waterproof Thermocouples	K-type	RS Pro
Thermocouple amplifiers	AD8495	Adafruit
3D-printer hot end heating blocks used like heating element and placed inside the water tank	3D-printer hot end heating blocks	MakerHawk
Level sensor	Self-made and coupled to an MPR121 capacitive sensor	Adafruit
Room Temperature and Humidity sensor	DHT22	Arduino
FEP sheet is used to seal the MI from the top, enabling UV light to pass through while preventing inner contamination when the sliding covers are not in place.	FEP film	3DJake
UV LEDs (278 nm)	LEUVA66B00HF00	LG Innotek
Heating glass	4mm thick NSG TEC™ 15	Yalos – Glass Technology
Pogo Pin connectors	811-22-007-30-001101 (male) 319-10-107-30-008000 (female)	Mill-Max Manufacturing Corp.
Glass temperature sensor	DS18B20	Maxim IC
Touch screen display	NX4024T032	Nextion

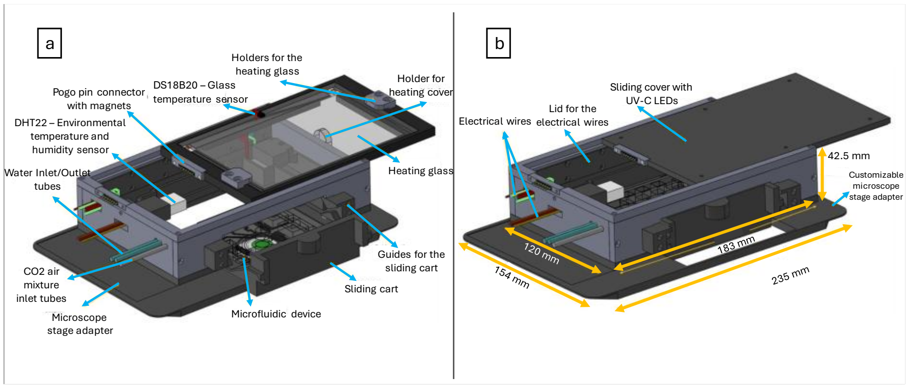

Figure S1. 3D CAD model of the designed MI. Two 3D CAD configuration concepts of the MI are shown in these figures. (a) Configuration utilized for cell incubation. The microfluidic device is placed inside the system thanks to the sliding cart. In the current view, the sliding lid with the heating glass is visible; the transparent heating glass on the top and the microfluidic device upon the sliding cart, inside the MI; (b) represents the configuration of the sterilization of the MI, previous to the biological experiments, with the UV cover inserted on top of the MI, instead of the heating glass.

Table S2. Detailed design dimensions of the microfluidic device of this study.

	**Width (mm)**	**Length (mm)**	**Height (mm)**	**Volume (μl)**	**Flow Rate (μl/h)**
Total dimension of the PMD	80	72	12		
Inlet/Outlet channel	0.250	300	0.100	7.5	11.2
Reservoir A, C	217.9 [mm^2]	6	1307.6		
Reservoir B	100 [mm^2]	6	600		
Reservoir D	780.25 [mm^2]	6	4681.5		
Waste Reservoir	1153.9 [mm^2]	2.7	3115.5		
Culture Chamber	3 (diameter)	2.2	15.5		

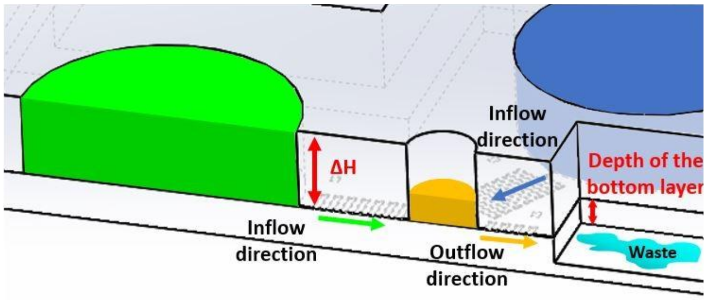

Figure S2. The concept of communicating vessels. Our devices are all open systems, meaning that they are exposed to atmospheric pressure. The reservoir containing media that are required to flow into target reservoirs, such as cell culture reservoirs, are filled until a certain height, so that the height discrepancy between the reservoirs is given by the difference of two heights. This difference gives rise to a pressure gradient (Stevino law) that is the driving force of the flow.

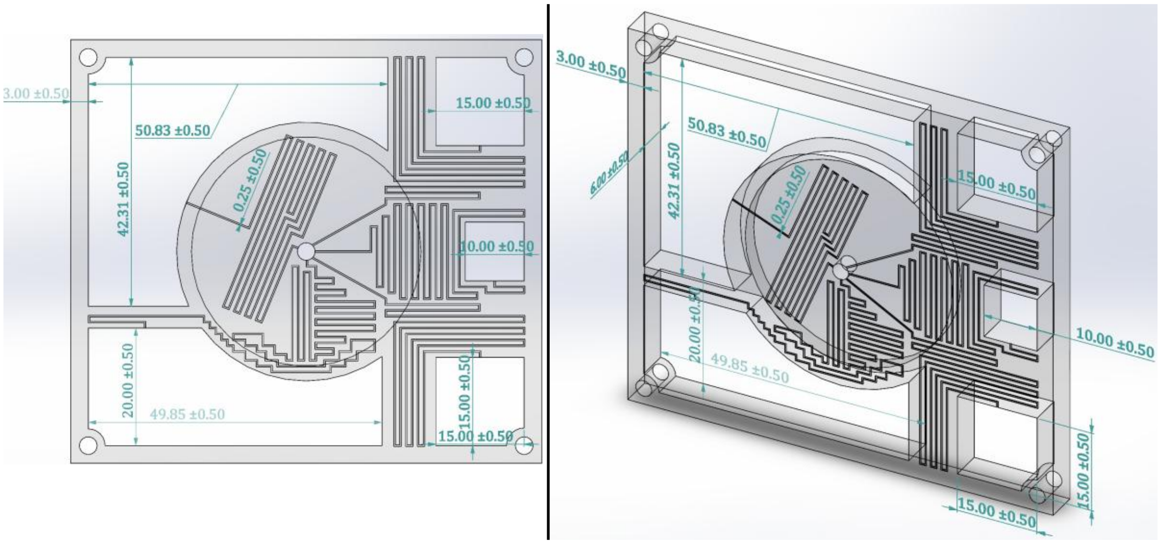

Figure S3. Detailed view of the channel layer. (left) front view; (right) trimetric view.

S1. FEM simulations

Following the CAD design, several computational simulations were implemented in Ansys Workbench 2021 R1 to predict the behavior of the system. Ansys CFX and Ansys Fluent were used as solvers. To reduce computational costs and improve simulation convergence, a simplified layout of the MI was adopted. To achieve this, only half of the geometry was simulated, and symmetry conditions were imposed to predict the behavior of the remaining half.

S1.1. Temperature analysis – water domain

The water inside the tank and the volume of the air inside the MI were considered as two separate fluid domains. Specifically, the simplified geometry used for the water tank is shown in Figure S3.a. The tank has a height of 14 mm, and it has inside the heating block 20 mm × 20 mm × 10 mm. A structured mesh was developed with a minimum element size of 2 mm and a maximum size of 4 mm. The mesh was optimized for CFD, with 7636 nodes and 6130 elements. The contribution of the heating glass and the thermal properties of the tank's material were not considered. The aluminum heating block was modelled as a surface heat

source placed on every wall in close contact with the water. Parameters used for aluminum are listed in Table S3. The water properties were imported and copied from the Fluent database instead. Five *Named Selections* were created and used for boundary conditions

- *Contact*, faces virtually in contact with the air domain (not considering the material of the tank);
- *Walls*, the bottom and back walls of the tank;
- *Heater*, the faces where heat source is in contact;
- *Symmetry*, the face with the symmetry condition;
- *Water*, the fluid domain.

Table S3. Parameters and values used in the simulation.

Parameter	Value	Description
Env_Temp	22 [°C]	Room temperature at t = 0 s
Glass_Temp	45 [°C]	Glass steady temperature at t = 0 s
Heater_Temp	60 [°C]	Heater cube steady temperature at t = 0 s
k_Glass 1	1,2 [W/m*K]	Glass thermal conductivity
k_Aluminium 2	202,4 [W/m*K]	Aluminium thermal conductivity
SH_Glass 1	750 [J/Kg*K]	Glass specific heat
SH_Aluminium 2	898.7 [J/Kg*K]	Aluminium specific heat
D_Glass 1	2230 [Kg/m^3]	Glass density
D_Aluminum [2]	2700 [Kg/m^3]	Aluminium density

Two different models were used for this simulation:

- Thermal energy, to activate the enthalpy/temperature equations, without considering the compressibility effects;
- Laminar flow, to model the flow.

Initially, at t=0 s, the temperature of the water and the outside air was set to 22 °C, while the heating block was set to 50 °C. The walls of the tank were assumed to be adiabatic. Moreover, although the heat transfer coefficient of a material varies with the temperature gradient, in these simulations, the heat transfer coefficient of the air was assumed to be constant and equal to $h_{ext} = 15\frac{W}{m^2\,K}$ [80].

In CFX solver, the heat flux, q, between water and air on the contact surfaces is computed as:

$$q = h_{ext} \cdot (T_{ext} - T_W)$$

Where h_{ext} is assumed to be the air heat transfer coefficient, T_{ext} is the external temperature (circa 296 K) and T_W is the wall surface temperature.

A transient study with a simulation time of 900 seconds was conducted. Since computational simulations usually diverge in the first time points, it is advisable to use a small time-step initially and gradually increase it as the resolution proceeds. Additional simulations were performed with the heating block temperature set to 70 °C and 80 °C.

Furthermore, the cooling process of the heating blocks was also simulated. This allowed for an investigation of what happens to the temperature of water when the hot heating blocks are turned off by the control system. In fact, it is crucial for the water temperature to not greatly exceed a specific target value, which was set to 37 °C for this simulation. Hence, three additional thermal balancing simulations were set, considering the water temperature set to 37 °C t=0 s and the heating blocks set respectively to 60 °C, 70 °C, and 80 °C for each simulation.

S1.2. Temperature analysis – air domain

Simplified geometry was used also for the air domain, and it is shown in Figure S3.b. The heating glass has been modelled as a heating surface at 45 °C placed on the top of the air volume and the material properties used are listed in Table S3. The thermal coupling between the air and water was modelled as a thermal input, utilizing a specific User Defined Function (UDF).

The air properties were imported from Fluent database. The water was defined as a "solid body" but with the same properties of the water liquid (imported from Fluent database) to be able to set-up the boundary conditions at the contact walls. A structured mesh was developed with a minimum element size of 2 mm and a maximum size of 4 mm. The mesh was optimized for CFD with 45544 nodes and 42881 elements. Five named selections were created and then used for the boundary conditions definition in the solver (Figure S3.b):

- *Glass*, the heating top face;
- *Contact*, the faces virtually in contact with the air domain (not considering the material of the tank);
- *Air*, the fluid domain;
- *Symmetry*, the face with the symmetry condition;
- *Walls*, the bottom and back walls of the tank.

Specifically, the heating profile obtained from the water domain simulation was used to simulate the thermal input of the water at the interface when it comes into contact with the air domain. At $t = 0\ s$, the initial temperature of the air was set at the room temperature value of 22 °C. A transient study was conducted, utilizing a second UDF to define the time-stepping strategy. The total simulation time was set to 820 s, with a varying time-step based on the UDF and a maximum of 20 iterations per step. Results were saved every 10 seconds.

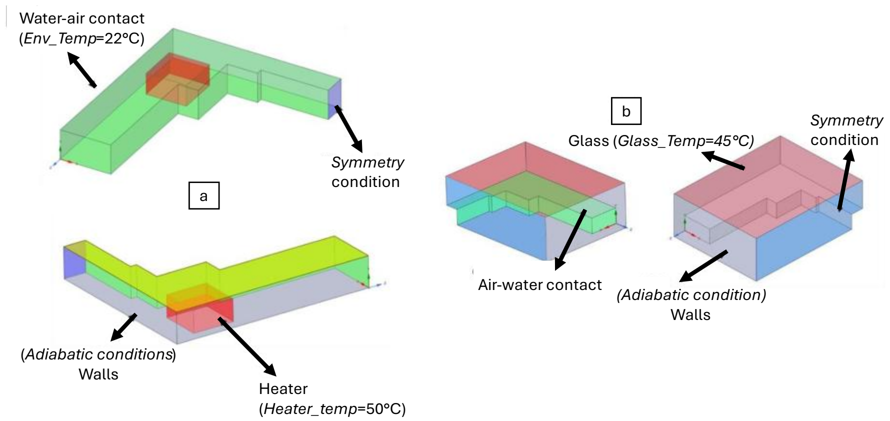

Figure S4. (a) Detailed view of the boundary conditions applied to the water domain. (b) Detailed view of the boundary conditions applied to the air domain. The UDF that models the thermal coupling is applied to the contact region between air and water.riga

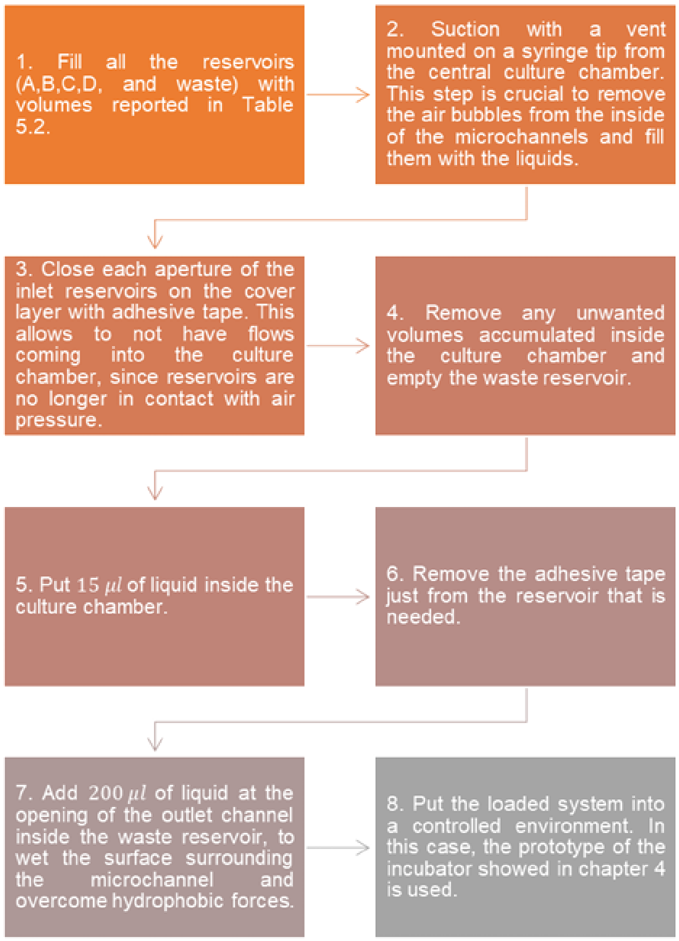

Figure S5. Flowrate measurement protocol. Steps 1-8 were repeated for each channel and the flowrate was evaluated by collecting the fluid volume from the waste reservoir to assess the effective flow rate of the microchannels of the designed platform. Precisely, the D reservoir was used to feed the cell culture, hence, it was evaluated in terms of the volume loss in time due to the flowrate.

S2. MI

The MI integrates three main systems for heating, humidification, and sterilization. Each system is controlled by a PID controller to ensure proper functioning.

S2.1. Sterilization system

Although culture media are usually enriched with antibiotics to counteract the proliferation of bacteria, a sterile environment is needed to limit any type of contamination. We designed a sliding sterilization lid mounted with 6 UV-LEDs at 278 nm, model LEUVA66B00HF00 produced by LG Innotek (Figure S5.a). The best number of LEDs to be used has been calculated based on the exposed area that needs to be sterilized. Moreover, we estimated that a few minutes are enough to deliver the proper energy to sterilize the system in our configuration. However, for thorough biological decontamination, the UV sterilization process lasted about 30 minutes before and after every experiment, ensuring a 99% reduction in contamination (Table S4). We experimentally evaluated the UV-C transparency of the FEP film. It is important to note that the farthest point inside the MI that needs to be sterilized is approximately 4-5 cm away from the UV-C light source. Our findings showed that the UV-sensitive negative photoresist can be developed through the FEP film within just 2 minutes, even when the sample is positioned 5 cm away from the light source. This proves the fact that the farthest distance of the MI to be sterilized is fully covered by our UV sterilization method.

S2.2. Heating system

The heating system consists of various components that contribute to reaching the target temperature of 37°C:

- Firstly, there is a heating glass measuring 10 mm × 15 mm × 4 mm (Figure S5.b), which is attached to one of the sliding lids. A contact mechanism using pogo pins and magnets allows the glass to be powered when it is positioned. This transparent glass enables the user to easily view the system without affecting the optical path of the microscope/spectroscope. However, its temperature cannot go above 45 °C when powered at 12 V. The PID control system maintains the temperature of the glass around the user-defined target value, and a dedicated sensor records the temperature;
- To increase the evaporation rate and relative humidity, two hot end heating blocks are placed inside the water tank, one on the right and one on the left. Each heating block measures 20 mm × 20 mm × 10 mm and can exceed a temperature of 200 °C. However, the PID control system prevents them from damaging the platform by limiting the temperature to the user-set target value. Waterproof thermocouples monitor the temperature of each heating block;
- Additional heaters can be positioned beneath the sliding cart (Figure S5.a), closer to the culture chamber. For the preliminary culture experiments, a simple custom-made donut-shaped Joule-effect heater was created using a conductive wire wrapped into Kapton sheets for electrical insulation. This design prevents accidental damage to users and unwanted short circuits. The central hole of the donut-shaped heater allows microscope/spectroscope objectives to approach the samples without affecting the optical path. A plug&play system is used to power the heater at 20 V and 4.5 A using a dedicated generator (Figure S5.c). This generator is connected to a programmable PID controller, which is external to the main PID control unit.

S2.3. Humidification system

The RH percentage in the environment is regulated by increasing or reducing the evaporation rate of the water tank, and by regulating the temperature of the heaters. As the water evaporates, the level inside the tank decreases over time. To address this, we have designed and dimensioned an automatic refill system.

The water is refilled or removed from the system through two small and flexible Tygon tubes that are connected to the water tank and pass via special holes manufactured on the left side panel of the MI (Figure S5.a). When a level sensor triggers the start signal, a volumetric pump automatically refills the tank with a certain amount of distilled and demineralized water. This process continues until the water level reaches a specific height and the sensor triggers the stop signal.

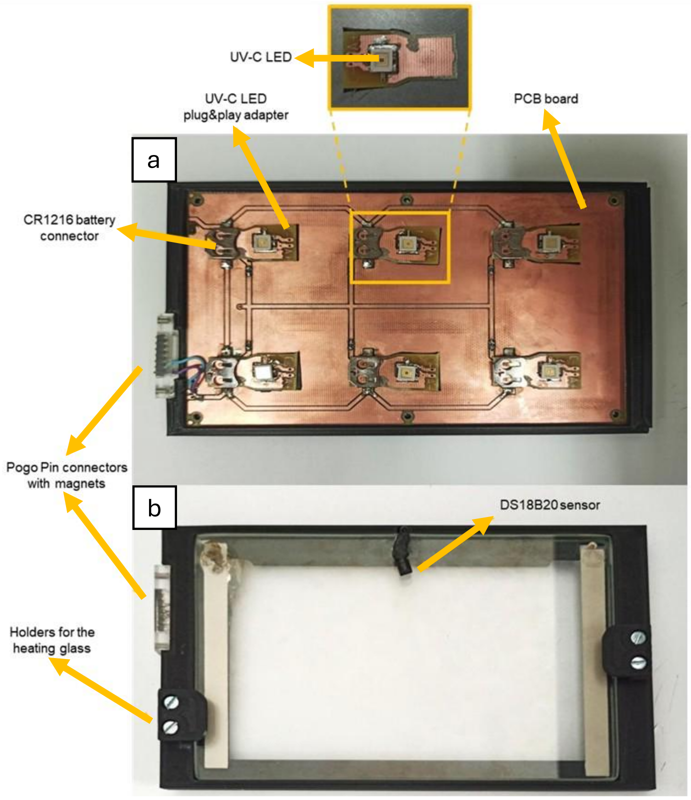

Figure S6. Top closures of the MI. (a) UV-C LEDs welded onto smaller PCB pieces and attached to the main PCB by means of plug&play connectors. (b) heating glass held in place inside the 3D-printed PLA frame.

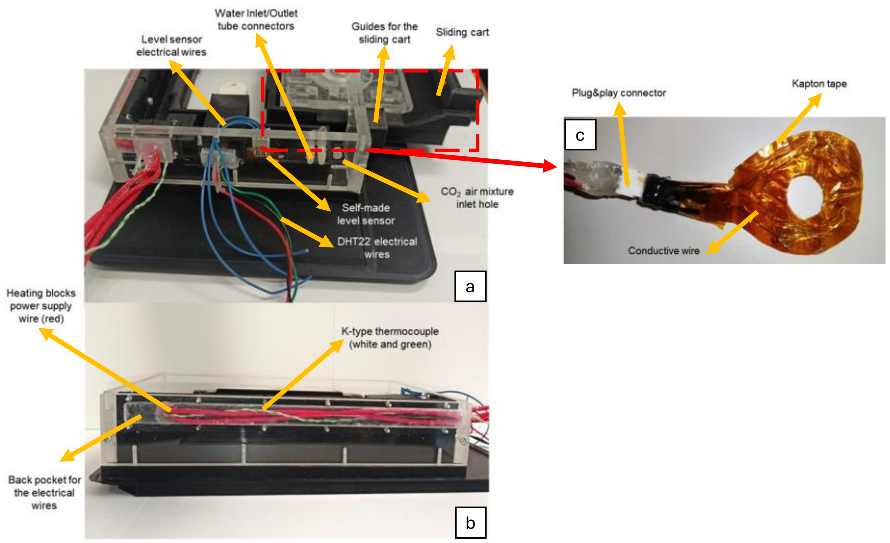

Figure S7. Side views of the MI. (a) Left side; (b) back; (c) heater attached beneath the sliding cart.

Table S4. Maximum UV-C dose $[mJ/cm^2]$ needed to inhibit different microorganism colonies formation at 253.7 nm. A single logarithmic reduction means a 90% kill factor, a double logarithmic reduction means a 99% of biological inactivation, while a triple logarithmic reduction means a 99.9% kill factor [81–83].

**Microorganism**	**UV Dose $[mJ/cm^2]$**		
	**Logarithmic reduction**		
	**1**	**2**	**3**
Spores (Bacillus anthracis spores)	24.32	46.2	
Bacteria (Pseudomonas stutzeri)	100.0	150.0	195.0
Yeasts (Saccharomyces spores)	8.0	17.6	
Molds (Aspergilius niger)	132.0	330.2	
Protozoa (Nematode Eggs)	45.0	92.0	
Viruses (Adenovirus type 40)	55.0	105.0	155.0

S2.4. Control and automation of the MI

The Teensy 3.2 microcontroller was used in this project. It was to the computer via USB port, and the recorded parameters can be viewed on a graphical user interface implemented in Python. The following information is received by the controller:

- The humidity and temperature values detected by DHT22 sensor;

- The temperature of the left and right heaters placed inside the water tank, measured by two K-type thermocouples;
- The glass temperature detected by the DS18B20 sensor;
- The status of the UV-C LEDs activation;
- The water level measured by the custom-made level sensor.

Real-time values are displayed on both the graphical user interface (GUI) and the LCD touchscreen attached to the top of the control unit while the platform is operating (Figure S7). The PID microcontroller processes all this data and manages the power distribution among all the electrical components with respect to the target values set by the user on the LCD display or on the GUI.

All the electrical wires inside the MI were placed into a specific pocket manufactured on the back panel of the system and covered with a specially designed lid that allowed them to go through silicone gaskets. This arrangement was implemented to reduce condensation on the electrical wires. The wires were routed externally until reaching the first dedicated case with DB9 and 12-pin circular connectors. From there, they were guided to a second dedicated casing which housed the microcontroller, LCD display, ON/OFF button, and USB port (Figure S8).

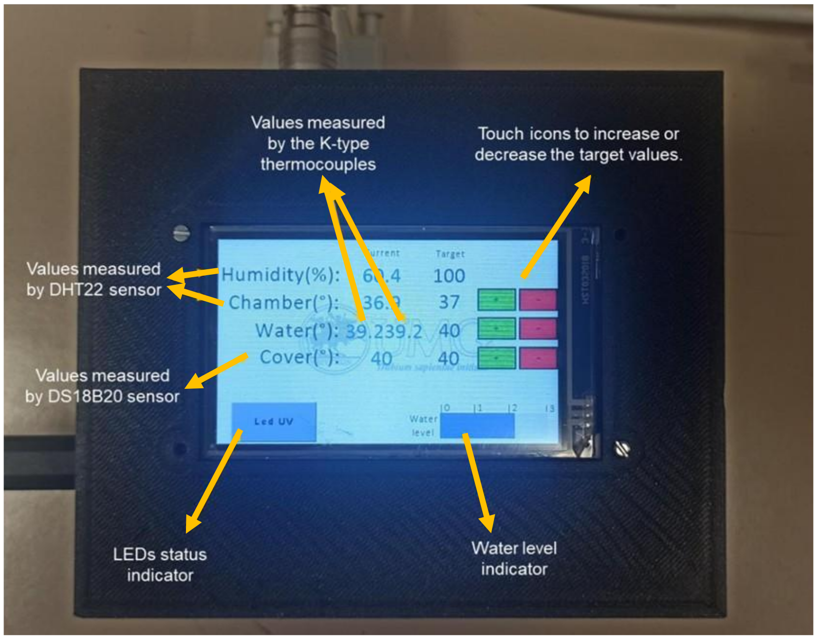

Figure S8. Touch screen display fixed onto the top face of the microcontroller case, showing in real-time several parameters of interest to the user. Target values can be also set up via the GUI developed in Python.

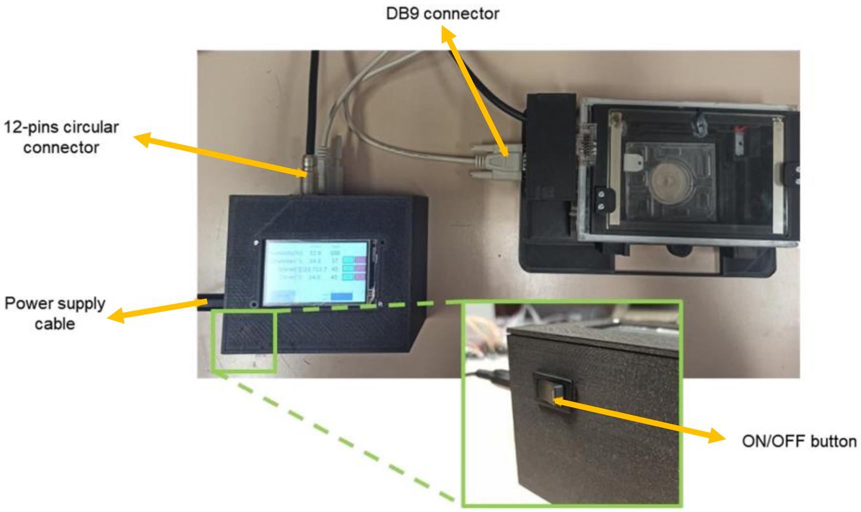

Figure S9. The assembled case of the control system. All the wires coming from the MI are collected into a dedicated housing placed on the left of the MI. Thanks to a DB9 connector and to a 12-pins circular connector, all the wires reach a second dedicated housing where the microcontroller and a touch screen display are placed.

S3. Hydraulic resistance determination criterium

It is well known that each type of microchannel has a parameter, k, which incorporates the geometrical characteristics of the cross-section that inevitably influence the flow:

$$R = k\eta L$$

The parameter k can be determined both analytically and numerically, by means of appropriate approximations of the Navier-Stokes equations. [57]. In the case of the rectangular shaped cross section channel, the hydraulic resistance is determined as follows:

$$R = \frac{12}{h^3 w(1 - 0.63\frac{h}{w})}\eta L$$

Where η is the viscosity of the cell-feeding medium, which is mainly composed of water, hence, it's viscosity may be approximated to be equal to $1 \cdot 10^{-3}$ Pa s at 37 °C, L the total length of the channel, h the height of the channel, and w the width of the channel.

Referring again to the supplementary table 2 and replacing the values, the theoretical hydraulic resistance can be determined as follows:

$$R = \frac{12 \cdot 1 \cdot 10^{-3} \cdot 300}{0.1^3 \cdot 0.25 \cdot (1 - \frac{0.63}{2.5})} Pa\, s\, m^{-3} \cong 1.92 \cdot 10^{13}\ Pa\, s\, m^{-3}$$

Table S5. Approximative cost of the microfluidic platform. The total cost is provided including man-work time.

**Component**	**Component/Model**	Cost
Microcontroller	Teensy 3.2	28.00 €
Waterproof Thermocouples (x3)	K-type	30.00 €
Thermocouple amplifiers (x3)	AD8495	20.00 €
3D-printer hot end heating blocks	-	19.00 €
Water Level sensor (self-made and coupled to a capacitive sensor)	MPR121	7.00 €
Room Temperature and Humidity sensor	DHT22	9.00 €
UV-permeable cover material (used to prevent contamination when covers are open)	FEP film	8.00 €
UV LEDs (278 nm wavelength) (x6)	LEUVA66B00HF00	200.00 €
Heating glass (4 mm thick)	NSG TEC™ 15	50.00 €
Pogo Pin connectors (male)	811-22-007-30-001101	12.39 €
Pogo Pin connectors (female)	319-10-107-30-008000	4.49 €
Glass temperature sensor	DS18B20	5.93 €
Touch screen display	NX4024T032	49.30 €

3D printing material (0.2 kg)	PLA	11.00 €
DC-DC Step Down Power Supply Module	XH-M133	5.00 €
DC-DC converter - 0.8-20V, 3A step-down	HW-133	3.00 €
PMMA (x3 100 cm^2), (13 euro x 1 m^2)	Rohm	0.50 €
PCB	-	6.90 €
Fabrication (15 euro/h) x 16 h	Man-work time	225.00 €
		**690.51 €**

216
