# Supplementary figures and images for: An Automated Microfluidic Platform for In Vitro Raman Analysis of Living Cells
Source: Biosensors (Basel). 2025 Jul 16;15(7):459. doi: 10.3390/bios15070459 (PMC12293637; doi:10.3390/bios15070459)

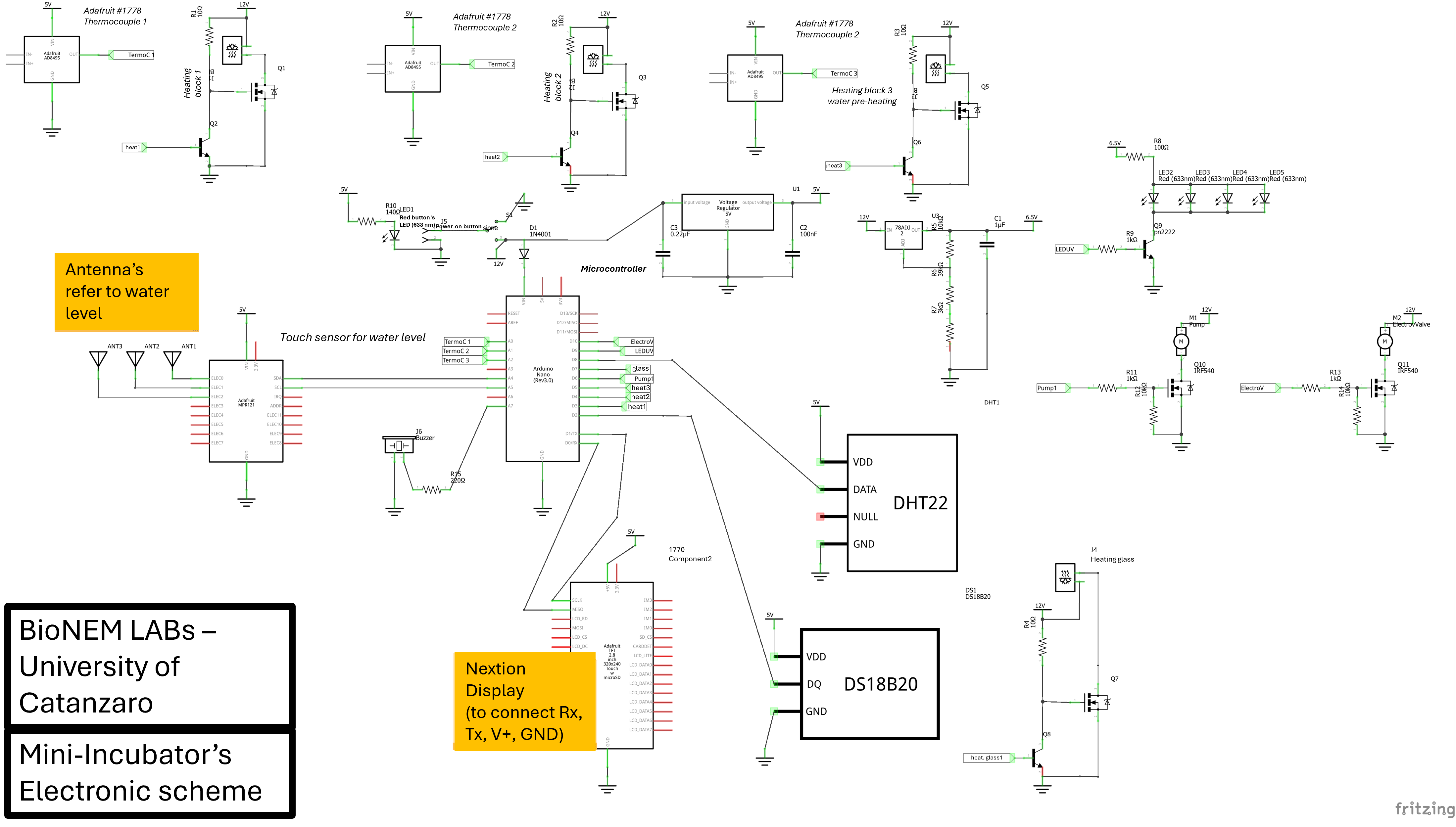

Supplement: Supplementary file 1 [file biosensors-15-00459-s001.zip › biosensors-3704532-supplementary/Mini-Incubator_electronic_scheme.png]
